# Level of awareness of Saudi medical students of the internet-based health-related information seeking and developing to support health services

**DOI:** 10.1186/s12911-020-01233-8

**Published:** 2020-09-03

**Authors:** Bader Aldebasi, Abdulaziz I. Alhassan, Sami Al-Nasser, Mostafa A. Abolfotouh

**Affiliations:** 1 King Abdullah International Medical Research Center, King Saud bin-Abdulaziz University for Health Sciences, Ministry of National Guard-Health Affairs, POB 22490, Riyadh, 11426 Saudi Arabia; 2 Department of Medical Education, College of Medicine, King Saud bin-Abdulaziz University, Riyadh, Saudi Arabia, Ministry of National Guard-Health Affairs, POB 22490, Riyadh, 11426 Saudi Arabia

**Keywords:** E-health knowledge, E-health perception, E-health application, E-health practice, IT methods, Medical informatics

## Abstract

**Background:**

Many studies are available in the literature about e-health in Saudi Arabia, however, data is limited to a few organizations and does not necessarily reflect the current and potential use of e-health for health care organizations in the country. This study aimed to determine the level of awareness of Saudi medical students of the internet-based health-related information seeking and developing to support health services and significant predictors of their practice.

**Methods:**

A cross-sectional survey of 440 medical students in Riyadh, Saudi Arabia, was conducted, during October/November 2019, using a previously validated questionnaire, to assess: (1) knowledge in three domains; e-health definition (13 statements), fields of application of e-health (8 statements), methods of using e-health (7 statements), (2) attitude toward using e-health (8 statements) and (3) reported practice of e-health in medical training (5 statements). A scoring system was used to calculate the total and percentage score of knowledge, attitude, and practice for each student. Multiple regression analysis was applied to identify predictors of e-health practice. Significance was considered at *p* < 0.05.

**Results:**

Of 440 medical students, the majority were females (55.7%) and from families whose monthly income was more than 10,000SR (82.8%). Overall knowledge about e-health was unsatisfactory (percentage mean score, PMS = 71.6%), with only 43.6% of students reporting a satisfactory level. However, this level was satisfactory for fields of application (Percentage mean score-PMS = 76.6%) and unsatisfactory for the definition of e-health (PMS = 70.7%) and methods of its use (PMS = 65.7%) domains. The overall attitude towards e-health use was positive (PMS = 82.3%), with nearly three-quarters of students (73.4%) reporting a positive attitude. Generally, a good level of practice of e-health was reported by students (PMS = 84.3%), with more than three-quarters of students (78.4%) reporting good practice. Adjusting for age, gender, stream, educational grade, and family monthly income, good practice was significantly predicted with higher knowledge (t = 2.22, *p* = 0.03) and attitude (t = 2.11, *p* = 0.04) scores.

**Conclusion:**

This study provides basic information regarding medical students’ level of awareness of internet-based health-related information seeking and developing to support health services. More resources should be directed to elevate medical students’ knowledge and to motivate them to practice e-health using the available tools.

## Background

Information technology (IT) methods in healthcare are well known to provide clinicians with health-related information and tools, such as clinical decision support systems. This software is expected to improve patient care and minimize medical errors [1]. In other words, the rising demand to improve healthcare processes via technical products and services has led to the tremendous development of wireless technologies worldwide [2].

E-health has been discussed widely in literature, workshops, scientific meetings, and the popular press. At the end of 2011, studies on e-health accounted for 1688 research data in PubMed, 18,600 in Google scholar, 2,420,000 in Yahoo search and 29,500,000 in Google.com [3]. Globally, the functions of e-health services have been expanding rapidly, intending to improve health, reduce costs of healthcare, increase equity and quality of healthcare, and improve communication between healthcare providers and patients [4].

There are many definitions of e-health, however, no universal agreement is there on any of them [3]. Moreover, there remains a lack of consistency in the use of e-health research [4]. A broad range of digital technologies and interventions used by stakeholders in many diverse settings have been described by e-health [5, 6]. E-health was defined as internet-based health-related information seeking and developing to support health services locally, regionally, and globally [7]. However, this definition was concerned with only patients seeking health information on the internet. E-health was defined by Shaw et al. [8] as an emerging field in the intersection of medical informatics, public health, and business. The term characterizes not only a technical development, but also a state-of-mind, a way of thinking, an attitude, and a commitment for networked global thinking, to improve healthcare locally, regionally, and worldwide by using information and communication technology. The World Health Organization recently defined e-health as “the combined use of electronic information and communication technologies (ICT) for health” [9]. This is an all-encompassing definition as it includes all aspects of health, from maintaining well-being to disease management.

In 2010, the World Health Organization called for more studies on e-health in developing countries [10]. E-health in Saudi Arabia is growing, as many organizational and individual initiatives have implemented e-health applications. Many studies are available in the literature about e-health in Saudi Arabia [10–34], however, data is limited to a few organizations and does not necessarily reflect the current and potential use of e-health for health care organizations in the country [35]. A descriptive analysis of the status of e-health in Saudi Arabia highlighted the national e-health initiatives such as the establishment of a new Master of Health Informatics degree program at King Saud bin-Abdulaziz University for Health Sciences (KSAU-HS) as an initiative to educate specialized professionals [11], with an e-health course to foster future professional development in this field in the country [15] and the role of the Saudi Association for Health Informatics (SAHI) in enhancing coordination among health information professionals [11]. In a survey to assess the level of usage of EHRs in government-related hospital in Saudi Arabia, showed only 16% use [17], and the major challenge facing full adoption of EHR in hospitals in Saudi Arabia was that most of physicians and nurses had negative attitudes toward EHRs relating to health information security and confidentiality concerns, lack of motivation or incentives to learn and use EHRs, and lack of sufficient training on using EHRs [17] Lack of e-health studies from the perspective of health managers and the limitation of studies to few geographical areas were identified by Alshhrani et al. [18] as knowledge gaps. The aim of the present study to determine the level of awareness of Saudi medical students at the college of medicine, King Saud bin-Abdulaziz University for Health Sciences (KSAU-HS), of the internet-based health-related information seeking and developing to support health services and significant predictors of their relevant practice, through the following objectives: 1) To determine the levels of knowledge about e-health, 2) To determine the level of attitude towards e-health use in medical practice, 3) To determine the field of practice of e-health, and 4) To identify the predictors of e-health practice among medical students.

## Methods

### Study setting

College of Medicine (COM-R), King Saud bin-Abdulaziz University for Health Sciences, Ministry of National Guard Health Affairs, Riyadh, Saudi Arabia. The Ministry of National Guard Health Affairs (MNGHA) serves the National Guard’s employees and their families and Saudi nationals in specific cases such as cancer patients. The MNGHA has installed systems and networks in all of its hospitals and it has implemented electronic medical record (EMR) systems, Picture Archiving and Communication Systems (PACS), and other systems.

### Study subjects

The study population is the students of the College of Medicine (COM-R), King Saud bin-Abdulaziz University for Health Sciences, Ministry of National Guard Health Affairs, Riyadh, Saudi Arabia. COM-R admits Saudi national mainly and awards a bachelor of medicine to two educational streams. Stream 1 has secondary school graduates and follows what is known as the conventional program. Stream 2 allows holders of Bachelor of Science degrees. These students follow what is known as the Graduate Entry Accelerated Program. This program is the first of its kind in the kingdom of Saudi Arabia and is designed to expedite the process of graduation by recognizing these students’ previous learning, thus, helping increase the market supply of physicians faster [12]. Medical students of both sexes, in both steams, during the study period will make the target of this study.

### Study design

A cross-sectional study to assess the levels of knowledge, attitude, and practice of medical students on e-health in the medical field. Students were contacted once.

### Sample size and sampling technique

Assuming a prevalence of 50% satisfactory level of knowledge of e-health among students, a confidence interval of 95%, and a margin of error of 5%, the estimated sample size was 384 students. To compensate for non-response of 20%, a total of 460 questionnaires were distributed to students of different educational grades, using an equal allocation method of sampling.

### Data collection

A previously validated self-administered questionnaire [36] was utilized to assess the awareness of medical students on e-health. Based on test re-test reliability, correlations for overall knowledge score, attitude score and practice score were: r = 0.89, r = 0.80 and r = 0.46 respectively. Internal consistency was high [Average Cronbach’s α = 0.80]. For this study, e-health was defined as “internet-based health-related information seeking and developing to support health services locally, regionally, and globally” [7]. However, this definition was concerned with only patients seeking health information on the internet. Thus, this survey was not designed to include telemedicine as a subset of e-health.

Knowledge: Knowledge about e-health was assessed in terms of 3 domains: what is e-health (13 statements), fields of its application (7 statements), and methods of its use (6 statements). Factual statements were responded by “yes”, “No” or “Do not know”. Knowledge score was calculated as follows: 1 point for a correct answer and 0 points for don’t know & wrong answer. The total score in each domain for each student was calculated by summing scores for all responses, and the overall level of knowledge was assessed by summing scores for all responses in the 3 domains, then a percentage score was calculated. This percentage score was categorized into satisfactory (> 75%) and unsatisfactory (≤75%).

Attitude: Attitude towards e-health was assessed in terms of; interest in receiving e-health training, use of patient electronic medical records in health settings, use of internet in health service/research, does the use of the computer by physician save time, should a computer be used in all health centers, does the use of computer relieve pressure on hospital outpatients, how it is difficult to use the internet in the field of health. Attitudinal statements were responded to by “strongly agree”, “agree”, “not sure”, “disagree” or “strongly disagree”. Attitude score was calculated by using a 5-point Likert scale ranging from 0 to 4 points; 4 points for strongly agree on positive attitude sentence, to 0 points for “strongly disagree”. The total score for each student was calculated by summing scores for all responses, and then a percentage score was calculated. This percentage score was categorized into positive (> 75%), neutral (50–75%), and negative(< 50%) attitudes.

The practice of e-health: Practice of students was assessed as regards: participation in a video conference, use of the internet for health-related information, and use of patient electronic records, program, and. Statements related to practice will be responded to by “No” or “Yes”. The practice score was calculated by giving a score of 1 for practicing and 0 for non-practicing. The total score for each student was calculated by summing scores for all responses, and then a percentage score was calculated. This percentage score was categorized into good (> 75%), average (50–75%), and poor (< 50%) levels.

The questionnaire includes questions on personal characteristics and previous experience in computer use. The investigator distributed an anonymous self-administered English based questionnaire inside an envelope, to ensure confidentiality, with a cover letter. Each envelope was handed to the student in his/her classroom. Students were expected to fill the questionnaire and return it in the envelope sealed with no identifiers. The cover letter served as the front page that explained the purpose of the study and invited the student to participate voluntarily. Agreement to fill the questionnaire was considered as a consent.

### Data analysis

SPSS software Ver. 25 was used for data entry and analysis. Descriptive statistics such as mean score and standard deviation, as well as frequency and percentages of all independent variables (age, gender, educational grade, etc. …), were used. Responses were scored by frequency and percentage then converted to percentage mean scores, then transformed to qualitative data as mentioned previously. To predict the significant predictors of student’s practice, multiple regression analysis was applied. Significance was considered at *p*-value < 0.05.

### Ethical considerations

The survey was introduced to the students in an envelope, with a cover letter, inviting the students to participate in the survey and explaining the aims of the study. They were informed that participation in the study was voluntary, that their agreement to participate would be considered as consent, and that their feedback would not affect their academic evaluation. The envelope was not recognized by the instructors. The collection of datasheets was framed with confidentiality in a matter where the student’s name, contact information, or badge number would not be identified or traced by anyone. This study was approved by the IRB of the Ministry of National Guard-Health Affairs [Ref. #SP18/488/R].

## Results

A total of 440 medical students were surveyed for their levels of awareness of e-health, the majority were females (55.7%), of stream 1 (79.8%), and of families whose monthly income was more than 10,000 SR (82.8%). Those students were distributed nearly equally on the four educational grades.

Table 1 shows the distribution of students according to their responses to factual statements on knowledge about e-health. Concerning the definition of e-health, the majority of students responded correctly to 11 statements, and incorrectly to 2 statements; with 65 and 58.6% of students respectively thinking that “patient examination through the use of internet” and “organization of health care for patients, including surgical operations, via the internet” did not constitute e-health. Concerning the fields of application of e-health, the majority of students correctly agreed on the following applications; clinical (80.5%), educational (89.5%), administrative (74.5%), policy-making (57.8%) and research (83.6%). Meanwhile, the majority agreed that video games and watching entertainment movies are applications of e-health (74.5%) & 73.9% respectively). Regarding the methods of using e-health, the majority of the students correctly agreed that visual communication (74.7%), internet (91.3%) and e-mail (81.1%) were methods of e-health, while around one-half of them agreed on paper-post (44.7%), fax (51.4%) and landline telephone (50.2%) as methods of using e-health.

**Table 1:** Response of medical students to factual statements on e-health

What is e-health?	Wrong (%)	Correct (%)
1 Health Care Online	25.4	74.6
2 Use of the Internet and other technology to improve health locally, regionallyand worldwide	9.1	90.9
3 Entertainment between working hours^a^	27.8	72.2
4 Shopping^a^	26.0	74.0
5 Social communication between friends^a^	28.9	71.1
6 Control of medicine supply for patients through electronic software	29.4	70.6
7 Provide medical advice to the patient through the use of the Internet	23.3	76.7
8 Patient examination through the use of the Internet	65.0	35.0
9 Organization of health care for patients, including surgical operations via theInternet	58.6	41.4
10 Provide electronic patient records	19.6	80.4
11 Monitor the health of the patient through electronic technologies	22.1	77.9
12 Education of doctors through the use of electronic sources	27.9	72.1
13 Exchange of medical information and medical communication between doctors	25.5	74.5
Percentage mean score	70.7 [unsatisfactory]	
**Fields of applications of e-health**	**No (%)**	**Yes (%)**
1 Clinical	19.5	80.5
2 Educational	10.5	89.5
3 Administrative	25.5	74.5
4 Policy maker	42.2	57.8
5 Research	16.4	83.6
6 Video games	25.5	74.5
7 Watching entertainment movies	26.1	73.9
Percentage mean score (%)	76.4 [satisfactory]	
**Methods of using e-health**	**No (%)**	**Yes (%)**
1 Visual communication	25.3	74.7
2 Paper post	55.3	44.7
3 Fax	48.6	51.4
4 Internet	8.7	91.3
5 Land line telephone	49.8	50.2
6 E-mail	18.9	81.1
Percentage mean score (%)	65.7 [unsatisfactory]	
**Overall Knowledge**	**no. %**	
Satisfactory	192 43.6	
Unsatisfactory	248 56.4	
Overall percentage mean score (%)	71.6 [unsatisfactory]	

^a^Wrong statements

The percentage mean scores for the three domains of knowledge about e-health, namely e-health definition (70.7%, unsatisfactory level of knowledge), fields of its application (76.4%, satisfactory level of knowledge), and methods of its use (65.7%, unsatisfactory level of knowledge). The percentage mean score of overall knowledge about e-health was 71.6% (unsatisfactory level). Only 43.6% of all students reported a satisfactory level of overall knowledge about e-health, Table 1.

Table 2 shows the responses of medical students to 8 attitudinal statements on using e-health. Generally speaking, the majority of students responded positively to the statements. However, less than two-thirds of them agreed or strongly agreed that “doctor use of a computer during patient interview saves time” (64.9%), and less than two-thirds disagreed that “the use of internet in the field of health is difficult”(63.2%). The percentage mean score of attitude towards the use of e-health was 82.32% (positive attitude). Nearly three-quarters of all students reported a positive attitude to e-health use (73.4%), with only 2% of them who reported a negative attitude.

**Table 2:** Response of medical students on attitudinal statements on using e-health

Statements	SA (%)	AG (%)	NS (%)	DA (%)	SD (%)
1. I think that the use of the Internet is useful in the field of health	85.1	12.0	2.5	0.2	0.2
2. I think that the use of the Internet in the field of health is difficult^a^	5.8	7.1	24.0	36.9	26.3
3. I think that doctor use of computer during patient interview saves time	37.2	27.7	22.4	8.3	4.4
4. I think doctor use of computer must be applied at all health centers	56.4	26.8	12.9	3.7	.2
5. I think that the use of computer relieves pressure on hospital outpatient	46.8	30.3	18.1	2.8	2.1
6. I think that patient’s electronic file must be applied in all centers and hospitals	73.9	16.9	7.9	1.2	0.1
7. I think e-Health can be used to increase awareness and control of communicable diseases	61.6	25.5	11.5	1.4	0.0
8. I am interested in receiving training for the use of electronic health	57.0	26.4	12.2	3.0	1.4
Overall attitude levels (%):	**Positive**		**Neutral**	**Negative**	
	73.4		24.6	2%	

^a^negative statement, *SA* strongly agree, *AG* agree, *NS* not sure, *DA* disagree, *SD* strongly disagree

Table 3 shows the forms of practice of e-health during medical training as reported by the medical students. Using electronic medical records and searching for health information ranked first (92.6% & 91.7% respectively), followed by using e-health in medical training (85.2%), while attending e-health training programs and participation in visual communication came last (77.5% & 74.1% respectively). The percentage mean score of overall practice was 84.3% (good level of practice). More than three-quarters of medical students (78.4%) reported good practice, only 9.1% of them reported poor practice.

**Table 3:** Practice of e-health during medical training among medical students

Practice	No (%)	Yes (%)
Searching for health information	8.3	91.7
Using electronic medical records during medical training	7.4	92.6
Attending e-health training programs	22.5	77.5
Using e-health in medical training	14.8	85.2
Participating in visual communication during medical training	25.9	74.1
Overall practice levels (%):		
Good	78.4	
Average	12.5	
Poor	9.1	
Overall percentage mean score (%)	84.3 [Good]	

Table 4 shows the results of a multiple regression analysis of e-health practice among medical students and the independent variables; gender, age, educational grade, stream type, monthly income attitude score, and knowledge score. It shows that higher e-health practice score was significantly predicted by higher knowledge (t = 2.22, *p* = 0.03) and attitude (t = 2.11, *p* = 0.04) scores, after adjusting for other variables in the regression model.

**Table 4:** Predictors of good e-health practice among medical students

Independent variables	B	SE	t-value	***p***-value
Constant	80.28	29.11	2.76	0.01
Gender	0.63	2.58	0.24	0.81
Age	−1.58	1.17	−1.35	0.18
Educational grade	1.49	1.54	0.97	0.33
Stream	−8.00	5.37	−1.49	0.14
Monthly income	3.72	2.25	1.65	0.10
Attitude score	0.23	0.11	2.11	0.04**
Knowledge score	0.18	0.08	2.22	0.03**

**statistical significance

## Discussion

It is believed that the use of information and communication technologies (ICT) can be a potential alternative to address some of the daunting problems in the healthcare sector in developing countries. Knowledge, skills, and acceptance of e-health by health professionals are essential attributes of successful integration of this new tool. In a study on medical students in Sri Lanka [37], 51 % rated their knowledge of e-health applications as minimal, and 43% were familiar with the term e-health. This finding was comparable with the findings of our study, where only 43.6% of all students reported a satisfactory level of overall knowledge about e-health. However, the fact that Sri Lanka’s study was conducted in 2007, while Saudi’s study was conducted during 2019, might affect the validity of the comparison between the findings of these two studies. This lack of knowledge on e-health could be attributed to the absence of formalized e-health educational components in the medical curriculum. On the other hand, nearly three-quarters of all students reported a positive attitude to e-health use, in our study. This finding was in agreement with the finding of a study in Sri Lanka [37], where over 80% believed that e-health had an important role to play in the current and future health sector, and another study in Bangladesh, where the majority of students believed the internet was useful for helping to make health decisions [38].

Medical educators recognize that electronic health record (EHR) proficiency is a skill required for students to succeed after medical school [39]. Medical students need hands-on experience documenting clinical encounters as well as entering orders to prepare for residency and become competent physicians. A 2012 survey of clerkship directors by the Alliance for Clinical Education and the University of Michigan Medical School also showed the limited scope of EHR use by students [40]. In the study of Sri Lanka, respondents had very poor access to computers and internet use was rare. In Bangladesh’s study of e-health literacy among university students [38], the majority of students used the internet to find health information, although a little over half of the students were competent in computers. However, in the present study, using electronic medical records and searching for health information, and using e-health in medical training were reported by almost all students, and attending e-health training programs and participation in visual communication was reported by three-quarters of students, with an overall good level of practice.

It has been reported that some factors such as; the level of education [38, 41–44], electronic device use [38, 43], advanced age [38, 43], and gender [43] influenced the use of the internet for health-related information [42, 43]. In Bangladesh’s study [38], e-health literacy was found to be influenced by age, education, and marital status, computer knowledge used to search for health information. In the present study, the e-health practice score was significantly predicted by both the knowledge and attitude scores, after adjusting for age, gender, stream type, educational grade, and family monthly income. This finding reflects the need for an educational e-health program for medical students.

This study has some limitations. Conduction of this survey on the medical students of one university would not allow for generalizing its conclusion. However, this study may open the door to investigating the challenges for upgrading the knowledge of e-health and the use of its applications by medical students in Saudi Arabia. The cross-sectional design of the study would not guarantee the causal association between the practice of e-health and the predictors of this practice among students. Longitudinal studies are recommended in the future to control for this limitation. Lastly, even though the tool of data collection is previously validated, it lacks the scope of telemedicine as a subset of e-health, and the absence of telemedicine is unfortunate, as clinical practice is what the students are trained for; using transmission of images of CT scans, ECGs and prescriptions by fax. However, this limitation could not be retrospectively rectified. Thus, conduction of further studies that include telemedicine and other aspects of e-health is necessary.

## Conclusion

Overall knowledge of students about e-health was less than satisfactory, while their attitudes were satisfactory and their practices were good. Students’ practice of e-health was significantly predicted by their levels of knowledge and attitude on e-health. This study could provide basic information regarding medical students’ knowledge, attitude, and practice on e-health.

More resources should be directed to elevate medical students’ knowledge and to motivate them to practice e-health using the available tools. It would help policymakers and education planners in developing appropriate evidence-based strategies and curriculum in medical and health institutes for successful implementation and use of e-health systems. Implementation of formalized e-health educational components in the medical curriculum is necessary, to provide knowledge about the fundamentals of e-health, basic concepts, and various applications. Such education must also include a practical component to provide medical students with necessary hands-on skills. Educational simulations without prior EHR training help students develop EHR competence [45].

## Data Availability

Most of the data supporting our findings are contained within the manuscript, and all others, excluding identifying/confidential respondent data should, will be shared upon request. Professor Mostafa Abolfotouh mabolfotouh@gmail.com (the corresponding author) could be contacted if someone wants to request the data.

## Abbreviations

PMS: Percentage mean score
COM-R: College of Medicine, Riyadh
KSAU-HS: King Saud bin-Abdulaziz University for Health Sciences
KAMC: King Abdulaziz Medical City
MNG-HA: Ministry of National Guard-Health Affairs
IRB: Institutional Review Board
ICT: Information and communication technologies
EHR: Electronic health records
EMR: Electronic medical records
IT: Information technology
PACS: Picture archiving and communication systems
SAHI: Saudi Association for Health Informatics
